# Behavioral alterations following blood-brain barrier disruption stimulated by focused ultrasound

**DOI:** 10.18632/oncotarget.8444

**Published:** 2016-03-28

**Authors:** Feng-Yi Yang, Sheng-Fang Huang, Irene Han-Juo Cheng

**Affiliations:** ^1^ Department of Biomedical Imaging and Radiological Sciences, National Yang-Ming University, Taipei, Taiwan; ^2^ Biophotonics and Molecular Imaging Research Center, National Yang-Ming University, Taipei, Taiwan; ^3^ Biomedical Engineering Research and Development Center, National Yang-Ming University, Taipei, Taiwan; ^4^ Institute of Brain Science, National Yang-Ming University, Taipei, Taiwan

**Keywords:** focused ultrasound, behavioral alterations, blood-brain barrier, memory, anxiety

## Abstract

The purpose of this study was to investigate the behavioral alterations and histological changes of the brain after FUS-induced BBB disruption (BBBD). Rats were behaviorally tested using the open field, hole-board, and grip strength tests from day 1 through day 32 after undergoing BBBD induced by FUS with either a mild or heavy parameter. In the open field test, we found an increase in center entries on day 1 and day 9 following heavy FUS treatment and a decrease in center entries at day 18 following mild FUS treatment. With regard to memory-related alterations, rats subjected to heavy FUS treatment exhibited longer latency to start exploring and to find the first baited hole. However, rats subjected to mild FUS treatment exhibited no significant differences in terms of memory performance or grip force. The obtained data suggest that heavy FUS treatment might induce hyperactivity, spatial memory impairment, and forelimb gripping deficits. Furthermore, while mild FUS treatment may have an impact on anxiety-related behaviors, the data suggested it had no impact on locomotor activity, memory, or grip force. Thus, the behavioral alterations following FUS-induced BBBD require further investigation before clinical application.

## INTRODUCTION

The blood-brain barrier (BBB) regulates the exchange of nutrients and waste between vasculature and brain tissue. At the same time, the BBB prevents the passage of larger molecules from the vasculature into the brain tissue [1]. Thus, most potent chemotherapeutics are ineffective in the brain diseases due to limited permeability of the BBB [2]. Several noninvasive methods have been developed to disrupt the BBB to facilitate the entry of therapeutic agents into the brain [3–5], but these methods can be problematic in that they enhance the delivery of drugs throughout the brain. Recently, focused ultrasound (FUS) with microbubbles has offered the potential to produce BBB disruption (BBBD) noninvasively in specific regions of the brain [6, 7].

It has been demonstrated that FUS-induced BBBD can be used to efficiently deliver systemically administered drugs to targeted brain regions in glioma-bearing mice, improving treatment efficacy [8, 9]. Moreover, the entire process of changes in BBB permeability induced by FUS can be quantitatively monitored by real-time dynamic contrast-enhanced magnetic resonance imaging (DCE-MRI) [10–12]. Thus far, the greatest limitation on the use of FUS-induced BBBD in clinical practice consists of safety concerns relating to cavitation in the brain. A real-time technique to monitor microbubble behavior to ensure safe BBBD is thus critical if FUS-induced BBBD is to be used in clinical applications.

Mechanical effects may be responsible for FUS-induced BBBD, but inertial cavitation could usually cause hemorrhaging or apoptosis in the brain tissue from the neighboring vessel. According to previous studies, however, when BBBD is performed using appropriate parameters, tissue damage can be avoided [7, 13]. Nevertheless, the acoustic intensities applied in BBBD are higher than those applied in transcranial diagnostic ultrasound. Although no significant negative effects resulting from histological examination, further evaluation of brain functions following FUS-induced BBBD were still needed. Such research will increase our knowledge of the behavioral alterations that occur following BBBD and allow for a better assessment of the safety of this technique in terms of its effects on brain functions.

The purpose of this study was to examine the impact of BBBD in terms of behavioral alterations following FUS exposure in the presence of microbubbles. Locomotor activity and habituation were assessed using the open field test, while memory impairments were evaluated using the hole-board test. In addition, forelimb gripping deficits were examined using the grip strength test.

## RESULTS

### Blood-brain barrier integrity and body weight change

**Figure 1:**
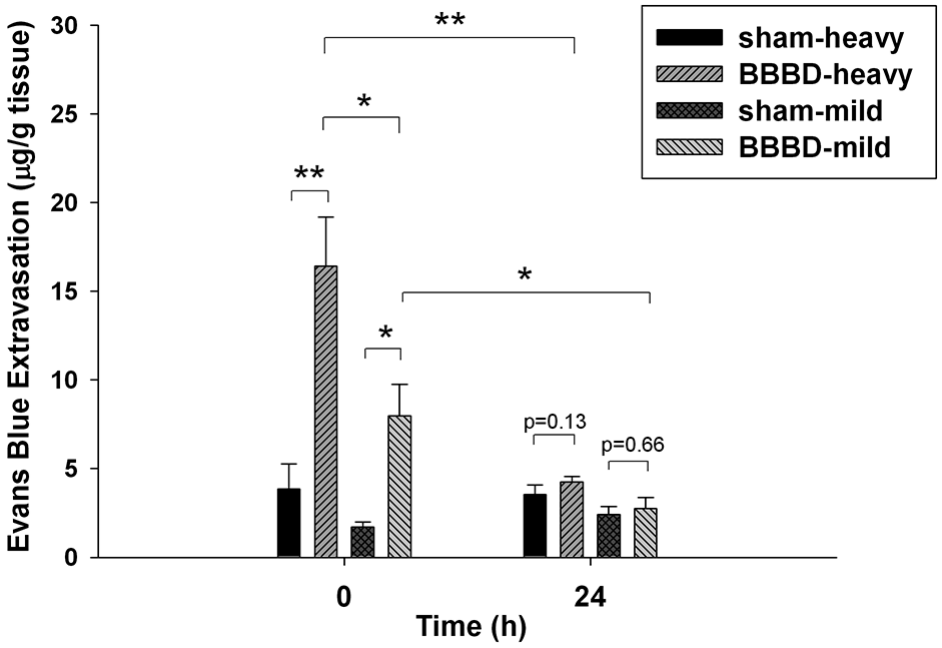
EB extravasation was quantified in the brain tissue immediately after and 24 h after sonication Immediately after heavy or mild sonication, the amount of EB extravasation in the sonicated brains was significantly higher than that in sham brains. Moreover, the amount of EB extravasation in the brains that underwent heavy sonication was significantly higher than in the brains that underwent mild sonication. No significant difference was found among them at 24 h following sonication. (*, *p* <0.05; **, *p*<0.01, *n* = 4).

Figure 1 shows the average extravasation of Evans blue (EB) (in μg/g of tissue) in brains injected intravenously with EB immediately and 24 h after heavy and mild sonication. In both cases, the amount of EB extravasation in the right sonicated brains was significantly greater than that in the right sham brains immediately after sonication. Moreover, the degree of EB extravasation in the brains subjected to heavy sonication was significantly higher than that in the brains that underwent mild sonication. Furthermore, in both cases BBB integrity (*p* > 0.05) appeared to have been re-established at 24 h based on the fact that administration of EB at this time led to no difference between the sonicated brains and its sham brain.

**Figure 2:**
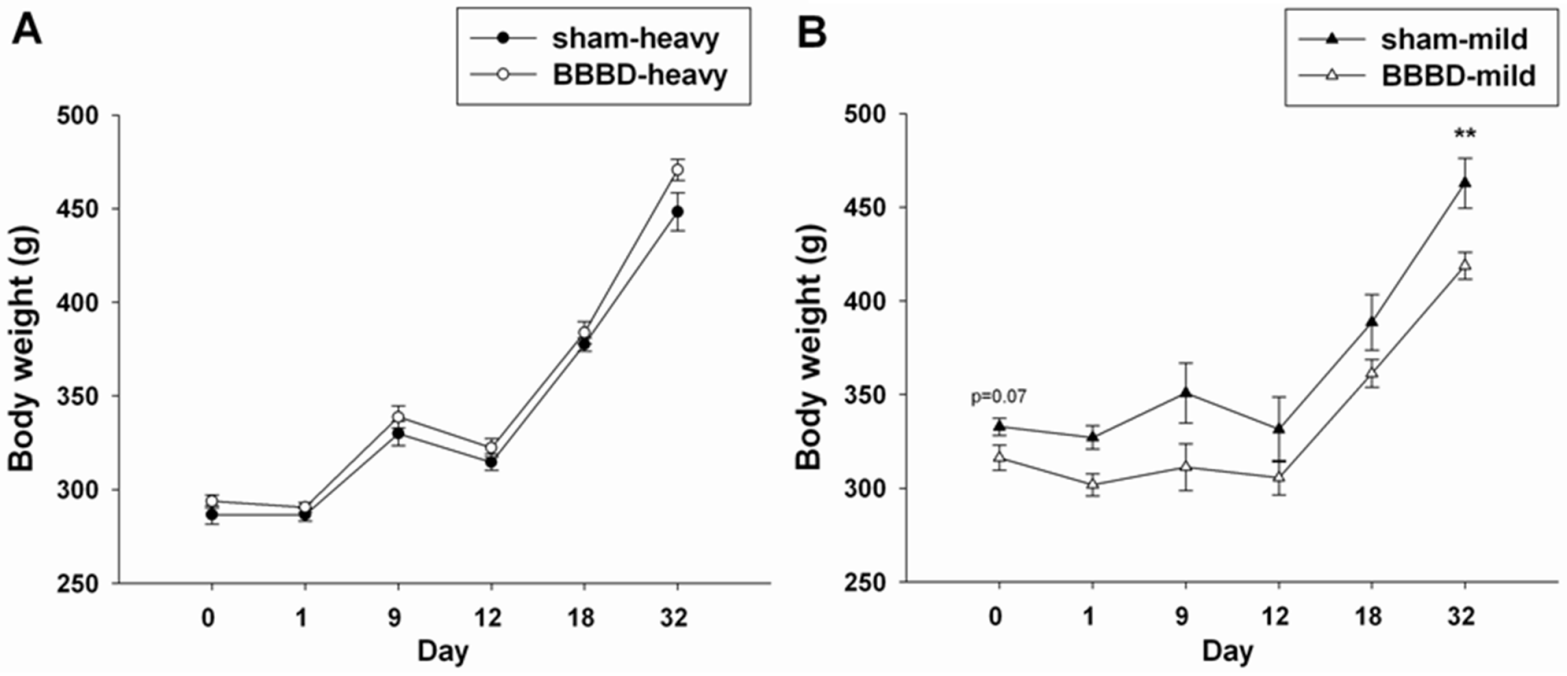
Body weight change after BBBD Graphs showing the the average body weights in **A.** the BBBD-heavy group and **B.** the BBBD-mild group on days 0, 1, 9, 12, 18, and 32 following sonication. * denotes significant differences compared to the sham group on day 32. (**, *p*<0.01, *n* = 8).

Body weight changes were recorded from day 0 to day 32 following heavy and mild sonication (Figure 2). No significant difference was found between the BBBD-heavy group and sham group at all time-points. However, there was a significant decrease in BBBD-mild group on day 32 after sonication.

### Open field activity

**Figure 3:**
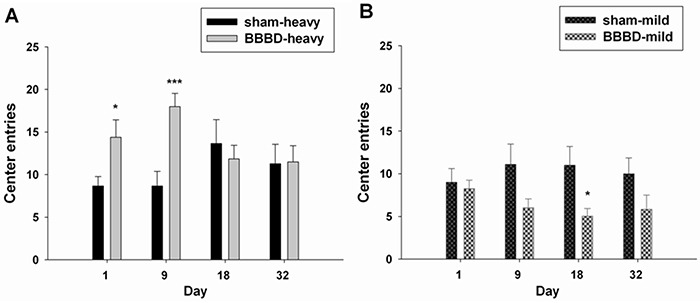
Effects of rats with FUS-induced BBBD on the open field activity **A.** The number of center entries was significantly increased in the BBBD-heavy group on days 1 and 9 following sonication. **B.** But the number of center entries was significantly decreased in the BBBD-mild group on day 18 following sonication. * denotes significant differences compared to the sham group at the same time points. (*, *p*<0.05; ***, *p*<0.005, *n* = 8).

**Figure 4:**
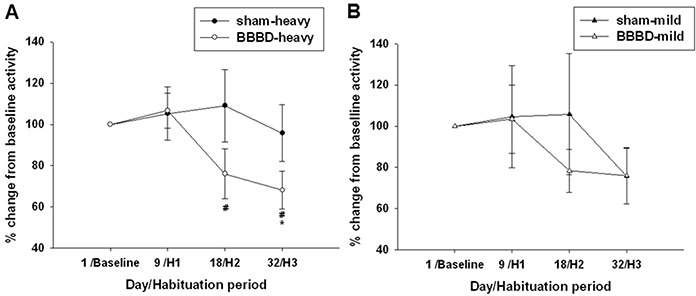
Habituation profile in the open field test on days 1, 9, 18, and 32 following sonication **A.** Habituation was observable in the heavy-BBBD rats, as highlighted by significant decreases in activity on day 32 (H3) relative to baseline activity. **B.** In contrast, mild-BBBD was not associated with significantly reduced activity levels at later time-points. * and ^#^ denote significant differences compared to day 1 (baseline) and day 9 (H1), respectively. (*,#, *p*<0.05, *n* = 8).

The open field test was performed to examine locomotor activity and habituation in the animals after FUS-induced BBBD. BBBD-heavy exposed rats entered the center of the open field significantly more frequently on days 1 and 9 post-sonication compared with sham group (Figure 3A). BBBD-mild exposure significantly reduced the number of center entries on day 18 post-sonication compared with sham group (Figure 3B). Figure 4 shows the habituation profile for global exploratory activity following heavy and mild BBBD. A significant decrease was observed between activity on day 32 (H3) and on day 1 (baseline) in the BBBD-heavy rats (Figure 4A). The activity levels on day 18 (H2) and day 32 (H3) were both significantly different than activity levels on day 9 (H1). No significant differences in habituation rates were found between any of the groups in BBBD-mild animals (Figure 4B).

### Hole-board test and grip strength test

**Figure 5:**
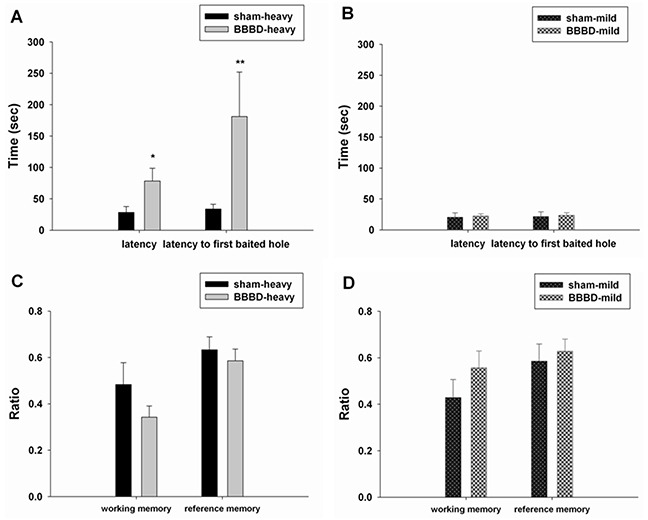
Hole-board testing was performed on day 12 following sonication The latency and latency to the first baited hole were recorded in **A.** the BBBD-heavy group and **B.** the BBBD-mild group. Working memory and reference memory were calculated in **C.** the BBBD-heavy group and **D.** the BBBD-mild group.* denotes a significant difference relative to sham group. (*, *p*<0.05; **, *p*<0.01, *n* = 8).

**Figure 6:**
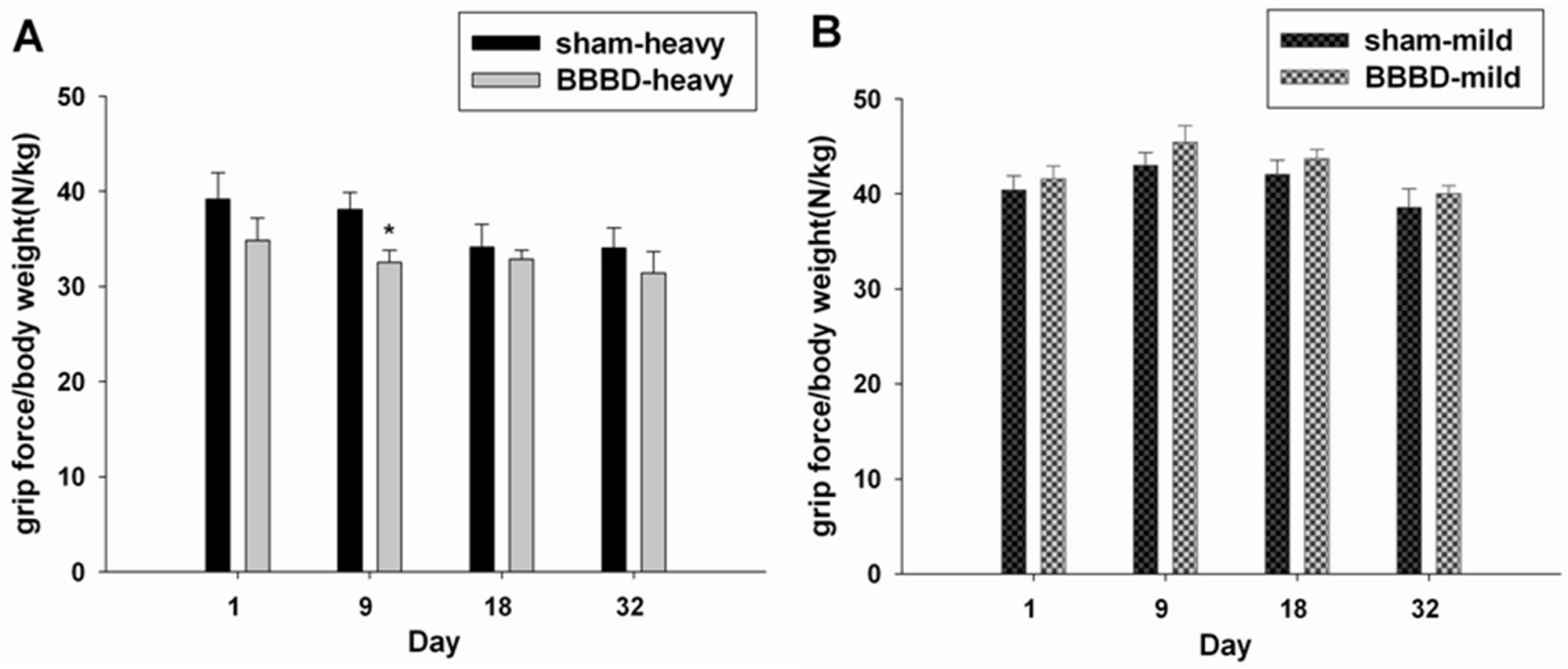
Effects of rats with BBBD on grip strength **A.** the BBBD-heavy group and **B.** the BBBD-mild group. * denotes a significant difference relative to sham group. (*, *p*<0.05, *n* = 8).

To evaluate the learning and memory ability, the hole-board teat was performed in the animals after FUS-induced BBBD. The BBBD-heavy group exhibited a significantly longer latency and latency to first baited hole compared with the sham group (Figure 5A). No significant differences in latency and latency to first baited hole were found between the BBBD-mild group and sham group (Figure 5B). In addition, no significant differences were observed between any of groups in terms of working memory or the reference memory rate (Figures 5C and 5D). Furthermore, Figure 6 shows the mean values of grip strength per body weight unit for the different BBBD groups. Grip strength was decreased significantly in the BBBD-heavy rats on day 9 post-sonication, but there was no significant difference in BBBD-mild group relative to sham groups.

### Histological observation

**Figure 7:**
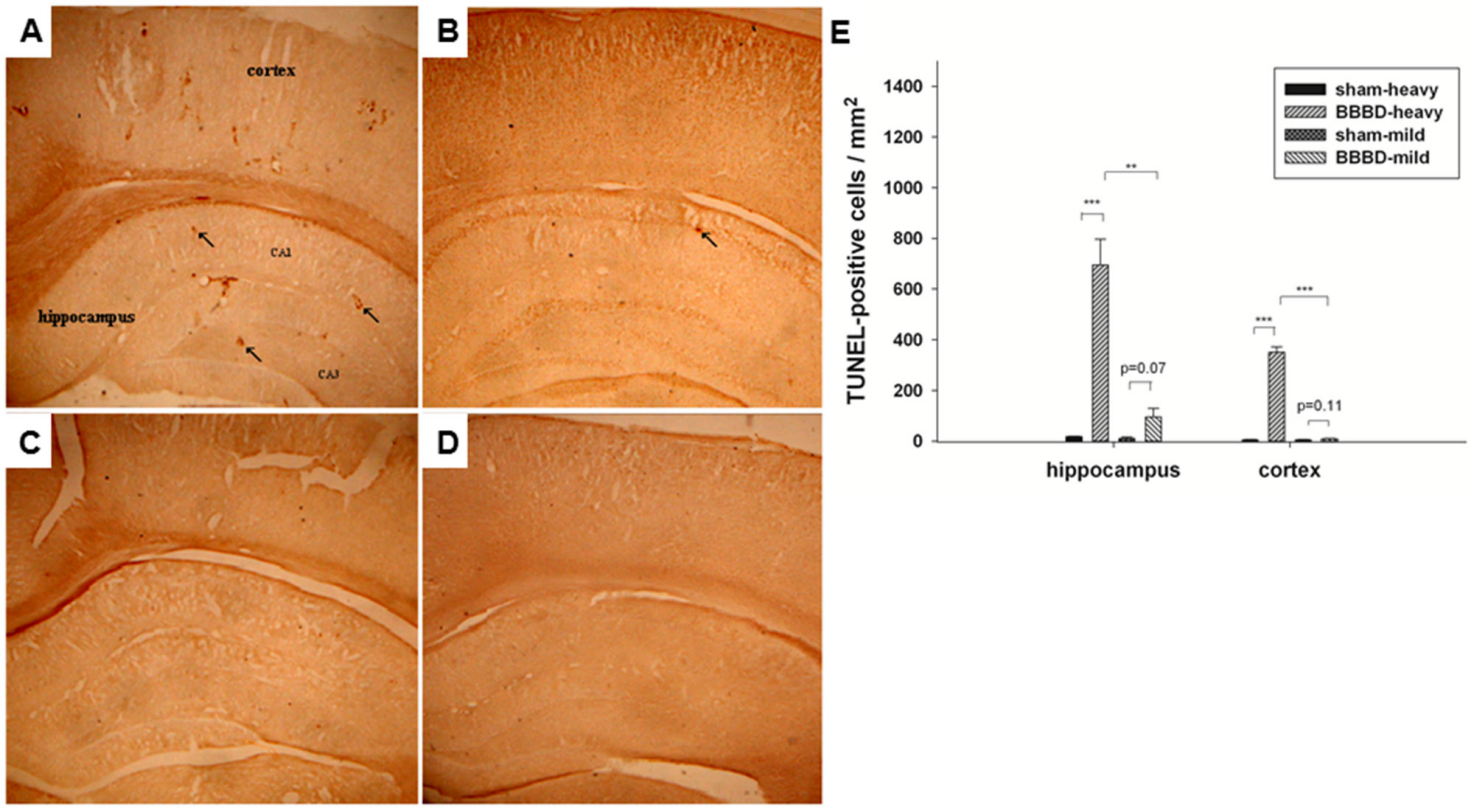
Effects of sonication on apoptotic cell death in the hippocampus and cortex in rats with BBBD Representative TUNEL-stained brain sections in **A.** the BBBD-heavy group and **B.** the BBBD-mild group on day 1 after sonication. **C.** and **D.** show the TUNEL staining on day 9 following sonicaton in the BBBD-heavy group and the BBBD-mild group, respectively. **E.** In the hippocampus and cortex, rats with mild-BBBD had fewer apoptotic cells than rats with heavy-BBBD on day 1 following sonication. Arrow indicates the apoptotic cells. * denotes a significant difference relative to sham group. (**, *p*<0.01; ***, *p*<0.005, *n* = 3).

**Figure 8:**
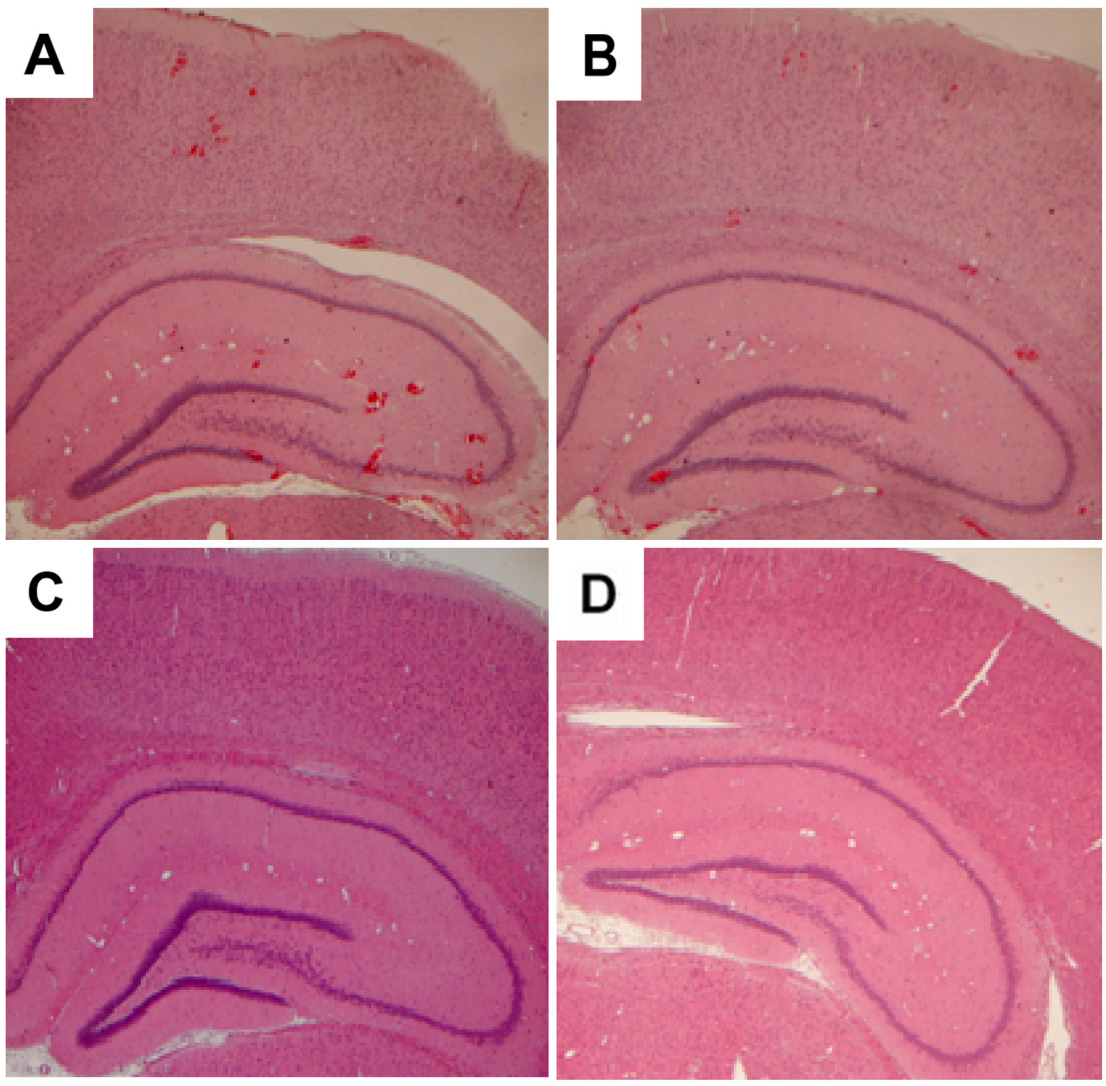
Observations of the brains with sonication by hematoxylin-eosin-stained sections Representative H&E-stained brain sections in **A.** the BBBD-heavy group and **B.** the BBBD-mild group on day 1 after sonication. **C.** and **D.** show the H&E staining on day 9 following sonicaton in the BBBD-heavy group and the BBBD-mild group, respectively.

As shown in Figure 7A and 7B, TUNEL-positive cells were observed in the BBBD-heavy and BBBD-mild groups on day 1 post-sonication. Moreover, fewer apoptotic cells were found in the BBBD-mild group compared with the BBBD-heavy group. No apoptotic cells were found in the sonicated brains subjected to heavy or mild BBBD on day 9 after sonication (Figures 7C and 7D). In the hippocampus and cortex, the number of apoptotic cells in the BBBD-heavy group was significantly higher than the number of apoptotic cells in the BBBD-mild group. However, no statistical differences of apoptosis were found in the BBBD-mild group in hippocampus and cortex part of brain (*p*<0.05). In addition, red blood cells were present in hippocampus and cortex part of brain, and were more severe for the BBBD-heavy group (Figures 8A and 8B). No hemorrhaging was observed in H&E staining of the brain on day 9 following BBBD (Figures 8C and 8D).

## DISCUSSION

To our knowledge, the current study is the first to explore the effect of BBBD induced by FUS on behavioral alterations in an animal model. The findings from this study indicate that BBBD-heavy rats produced hyperactivity and habituation deficit relative to sham animals under open field test. Moreover, the BBBD-heavy rats revealed spatial memory impairment in the hole-board test and a forelimb gripping deficit in the grip strength test compared to sham group. In contrast, BBBD-mild rats only exhibited anxiety-related behaviors in the open field test.

FUS-induced BBBD is a promising method by which to enhance the delivery of drugs to target sites for the treatment of brain diseases. Damage to the BBB, however, can alter brain homeostasis or neuronal activity because BBBD may allow harmful substances into the brain. It has previously been shown that acute, transient BBBD produces behavioral and electrographic seizures that correlate with the degree of BBBD [14]. A focal BBBD causes reactive changes in the brain that lead to abnormal excitability [15]. Therefore, further investigations are required to discover potential side effects on brain functions before FUS-induced BBBD is applied in routine clinical applications. In this study, the BBB was disrupted immediately after FUS exposure and recovered at 24 h following BBBD. The extent of BBBD was dependent on the ultrasound parameters. From histological observations, it could be seen that the occurrence of hemorrhaging and apoptosis were consistent with the degree of BBBD. The results suggest that the integrity of the BBB and the damage of brain tissue could be recovered after FUS exposure.

The real mechanisms of FUS-induced BBBD are still unknown. Among the most likely causes are the mechanical effects associated with interactions between acoustic waves and microbubbles. Previous studies have indicated that vasoconstriction might be related to BBBD during FUS exposure [16, 17]. This constriction might result in temporary ischemia, which can induce BBBD by modifying cerebral blood flow. Most studies that have used animal behavioral assessments have shown that ischemia is associated with subsequent hyperactivity and habituation deficits [18, 19]. The data obtained in the present study indicated that heavy BBBD induced by FUS resulted in hyperactivity and habituation deficits during the open field test. This was taken to indicate hyperactivity and impaired habituation, possibly resulting from transient ischemia induced by FUS. In addition, Figure 3B indicates that mild BBBD in rats resulted in anxiety-like behavior on day 18 following FUS exposure. This might explain why the average body weight in the BBBD-mild rats was significantly decreased on day 32 post-sonication compared with sham group due to a poor appetite.

Moderate to severe loss of hippocampus typically produces spatial memory and learning impairments [20, 21]. Figure 5 indicates that heavy BBBD can induce deficits in spatial memory without affecting working memory and reference memory. One explanation of this result is that the BBBD-heavy animals, because of hippocampal damage, were impaired in terms of forming spatial maps of their environments. Such a spatial memory deficit would be consistent with the significant apoptosis in the hippocampus indicated by TUNEL staining. Furthermore, grip strength was decreased significantly (*p*<0.05) in rats subjected to heavy BBBD on day 9 as compared to shams. As shown in Figure 7, there was also significant apoptosis in the cortices of the BBBD-heavy rats. This observation is consistent with a previously published study, which showed that unilateral lesions of the sensorimotor cortex do lead to a loss of gripping ability of the contralateral forepaw [22].

Several studies have shown that stimulation with low-intensity pulsed ultrasound (LIPUS) increases the levels of neurotrophic factors in the hippocampus of the brain [23, 24]. Moreover, another report demonstrated that the neuroprotective effects induced by LIPUS against brain injury in the sonicated brain [25]. The permeability and time window of FUS-induced BBBD can be effectively modulated with LIPUS. LIPUS also significantly reduced hemorrhages, neuronal death, and apoptosis in the sonicated brain. As a safe and effective neuroprotection strategy, LIPUS might be proposed as an adjunct treatment modality for brain injuries after FUS-induced BBBD in the future clinical applications.

FUS-induced BBBD represents a major advance in the targeted drug delivery of the brain. The current study suggests the possibility that manipulation of FUS-induced BBBD might produce a variety of behavioral changes after enhanced drug delivery. Further investigations of behavioral alterations in animals following FUS-induced BBBD are needed in order to avoid abnormal brain functioning in humans following future clinical uses of FUS-induced BBBD.

## MATERIALS AND METHODS

### Animals

All procedures involving animals were conducted in accordance with the guidelines for the Care and Use of Laboratory Animals. The study protocol was approved by the Animal Care and Use Committee of National Yang Ming University. Male Sprague-Dawley (SD) rats weighing from 300 to 350 g were used in this study. The animals were housed in groups of four in cages placed in a room on a 12:12 h light-dark cycle in which the temperature was maintained at 24 ± 1°C. All the rats had free access to food and water. Before FUS sonication, each animal was anesthetized intraperitoneally with chloral hydrate (400 mg/kg), and the body temperature was maintained at 37°C using a heating pad. The top of the cranium was shaved and the scalp overlying the skull was incised to facilitate the use of the bregma of the rat skull as an anatomic landmark for targeting.

### Focused ultrasound setup and sonication

The ultrasound system and equipment setup were the same as used in our previous study [26]. FUS exposure was generated by a 1 MHz single-element focused transducer (A392S, Panametrics, Waltham, MA, USA) with a diameter of 38 mm and a radius of curvature of 63.5 mm. The half-maximum of the pressure amplitude of the focal zone had a diameter and length of 3 mm and 26 mm, respectively. The transducer was mounted on a removable cone filled with deionized and degassed water whose tip was capped by a polyurethane membrane, and the center of the focal zone was about 5.7 mm away from the cone tip. FUS exposure was precisely targeted using a stereotaxic apparatus (Stoelting, Wood Dale, IL, USA) that utilized the bregma of the skull as the anatomical landmark. The rat's head was mounted on the stereotaxic apparatus with the nose bar positioned 3.3 mm below the interaural line. Ultrasound transmission gel (Pharmaceutical Innovations, Newark, NJ, USA) was used to cover the area between the transducer and the rat's skull in order to maximize the transmission of the ultrasound. Pulsed FUS was applied with a burst length of 50 ms at a 5% duty cycle and a repetition frequency of 1 Hz. The FUS was delivered to the targeted region in the right hemisphere of the brain located 3.5 mm posterior and 2.5 mm lateral to the bregma, and 5.7 mm below the skull surface. The ultrasound contrast agent (UCA) (SonoVue, Bracco International, phospholipid-coated microbubbles with mean diameter = 2.5 μm and concentration = 1–5 × 10^8^ bubbles/ml) was intravenously administered via the femoral vein approximately 15 s before sonication. In the animal experiments, rats were randomized into four groups (sham-heavy, BBBD-heavy, sham-mild, and BBBD-mild) for behavioral assessment and histological examination. After receiving the UCA at 450 μl/kg, the BBBD-heavy and sham-heavy rats were treated with and without sonication at an acoustic power of 5.72 W (corresponding to peak-negative pressure of 1.2 MPa), respectively. After receiving the UCA at 150 μl/kg, the BBBD-mild and sham-mild rats were treated with and without sonication at an acoustic power of 2.86 W (corresponding to peak-negative pressure of 0.7 MPa), respectively.

### Assessment of blood-brain barrier permeability

The BBB disruption (BBBD) can be quantified based on the extravasation of EB, which binds to albumin. This has been used previously for the evaluation of vascular permeability induced by focused ultrasound [27]. In this study, we used EB to assess the relationship between behavioral alterations and BBB integrity after FUS sonication. The rats were injected intravenously with EB (Sigma, St. Louis, MO) at a concentration of 100 mg/kg at 0 and 24 hours after FUS application. The animals were sacrificed approximately 4 hours after the EB injection. Animals were perfused with saline via the left ventricle until colorless perfusion fluid appeared from the right atrium. After perfusion and brain removal, the brain was sectioned into three slices from 0 to 6 mm posterior to the bregma and these were mounted on glass slides. The coronal sections were then divided into right and left hemispheres before the amount of EB extravasated was measured. Samples were weighed and then soaked in 50% trichloroacetic acid solution. After homogenization and centrifugation, the extracted dye was diluted with ethanol (1:3), and the amount of dye was measured using a spectrophotometer (PowerWave 340, BioTek, USA) at 620 nm. The content of EB in the tissue was quantified using a linear regression standard curve derived from seven concentrations of the dye, and was denoted per gram of tissue.

### Open field activity

Activity in the open field was tested with the automated Flex-Field/Open field Photobeam Activity System on post-sonication days 1, 9, 18, and 32. The system consisted of clear plastic chambers (41 × 41 × 41 cm), a PC interface board, and a computer for the recording and analysis of data. Two sensor frames, each consisting of a 16 × 16 photobeam array, were placed at 1.5 cm and 6 cm above the bottom of the chambers, respectively, and were used to detect movements in the horizontal and vertical planes. Each rat was placed in the center of the field. During a 5-min observation period, the number (center entries) and distance (center entries) of center square crossed, and total distance were recorded [28, 29].

### Hole-board assay

Spatial learning ability was studied by means of a hole-board apparatus in which food rewards were used as positive motivation. The modified hole-board consisted of a gray PVC box (50 × 50 × 50 cm) on which 16 holes (2.9 cm in diameter) were arranged in four lines. Prior to the hole-board testing, the animals were deprived of food for 48 h. On day 12 following sonication, six of the holes were baited with a small piece of food. A hole visit was scored when the rat introduced its nose into a hole. The latency to obtain the reward and the number of repeat visits were recorded. The latency was defined as the time from the beginning of the run to the entry into the first hole. The latency to the first baited hole was defined as the time from the beginning of the run to the time when the animal placed its nose into the first baited hole. The reference memory ratio was expressed as the number of visits or revisits to the baited holes divided by the total number of visits and revisits to baited and non-baited holes. The working memory ratio was expressed as the number of food-rewarded visits to the number of visits and revisits to the baited holes [30, 31].

### Grip strength

Neuromuscular strength was tested with the grip strength test on days 1, 9, 18, and 32 post-sonication. A grip strength meter (O'Hara & Co., Tokyo, Japan) was used to assess forelimb grip strength. Rats were lifted and held by their tail so that their forepaws could grasp a wire grid. The rats were then gently pulled backward by the tail with their posture parallel to the surface of the table until they released the grid. The peak force applied by the forelimbs of each rat was recorded in Newtons (N). Each rat was tested three times, and the greatest measured value was used for statistical analysis [32].

### Histology

Three rats from each group were prepared for histological observation. The rats were perfused with saline and 10% neutral buffered formalin on days 1 and 9 after the FUS sonication. The brains were removed and embedded in paraffin and then serially sectioned into 6-μm thick slices. The slices were stained with hematoxylin-eosin (H&E) to visualize their general cellular structure. Moreover, the slices were stained by TUNEL staining (DeadEnd Colorimetric TUNEL system, G7130, Promega, Madison, WI, USA) in order to detect DNA fragmentation and apoptotic bodies within the cells. The histological examination was carried out by light microscopy (Olympus BX61, Olympus, Shinjuku-Ku, Tokyo, Japan). A total of 6 tissue sections from each brain were analyzed for each animal. The areas showing apoptosis were measured using the Image-Pro Plus software (Media Cybemetics, Silver Spring, MD) in a blinded manner. TUNEL-positive cells were expressed as cells per unit area of the field.

### Statistical analysis

All values are shown as means ± SEM. The behavioral assessment data were analyzed using the Mann-Whitney U test. Other data were analyzed using the unpaired Student's *t* test. Statistical significance was defined as a *p* value ≤ 0.05.

## References

[1] Pardridge WM (2005). The blood-brain barrier: bottleneck in brain drug development. NeuroRx.

[2] Cordon-Cardo C, O'Brien JP, Casals D, Rittman-Grauer L, Biedler JL, Melamed MR, Bertino JR Multidrug-resistance gene (P-glycoprotein) is expressed by endothelial cells at blood-brain barrier sites.

[3] Nacucchio MC, Gatto Bellora MJ, Sordelli DO, D'Aquino M (1988). Enhanced liposome-mediated antibacterial activity of piperacillin and gentamicin against gram-negative bacilli in vitro. J Microencapsul.

[4] Pardridge WM (2002). Drug and gene delivery to the brain: the vascular route. Neuron.

[5] Pardridge WM (2003). Blood-brain barrier genomics and the use of endogenous transporters to cause drug penetration into the brain. Curr Opin Drug Discov Dev.

[6] Hynynen K, McDannold N, Vykhodtseva N, Jolesz FA (2001). Noninvasive MR imaging-guided focal opening of the blood-brain barrier in rabbits. Radiology.

[7] Hynynen K, McDannold N, Sheikov NA, Jolesz FA, Vykhodtseva N (2005). Local and reversible blood-brain barrier disruption by noninvasive focused ultrasound at frequencies suitable for trans-skull sonications. Neuroimage.

[8] Yang FY, Teng MC, Lu M, Liang HF, Lee YR, Yen CC, Liang ML, Wong TT (2012). Treating glioblastoma multiforme with selective high-dose liposomal doxorubicin chemotherapy induced by repeated focused ultrasound. International journal of nanomedicine.

[9] Yang FY, Wong TT, Teng MC, Liu RS, Lu M, Liang HF, Wei MC (2012). Focused ultrasound and interleukin-4 receptor-targeted liposomal doxorubicin for enhanced targeted drug delivery and antitumor effect in glioblastoma multiforme. Journal of controlled release.

[10] Choi JJ, Pernot M, Brown TR, Small SA, Konofagou EE (2007). Spatio-temporal analysis of molecular delivery through the blood-brain barrier using focused ultrasound. Physics in medicine and biology.

[11] Huang SY, Ko CE, Chen GS, Chung IF, Yang FY (2014). Focused ultrasound simultaneous irradiation/MRI imaging, and two-stage general kinetic model. PloS one.

[12] Yang FY, Ko CE, Huang SY, Chung IF, Chen GS (2014). Pharmacokinetic changes induced by focused ultrasound in glioma-bearing rats as measured by dynamic contrast-enhanced MRI. PloS one.

[13] Yang F, Fu W, Yang R, Liou H, Kang K, Lin W (2007). Quantitative evaluation of focused ultrasound with a contrast agent on blood-brain barrier disruption. Ultrasound in medicine & biology.

[14] Marchi N, Angelov L, Masaryk T, Fazio V, Granata T, Hernandez N, Hallene K, Diglaw T, Franic L, Najm I, Janigro D (2007). Seizure-promoting effect of blood-brain barrier disruption. Epilepsia.

[15] Seiffert E, Dreier JP, Ivens S, Bechmann I, Tomkins O, Heinemann U, Friedman A (2004). Lasting blood-brain barrier disruption induces epileptic focus in the rat somatosensory cortex. The Journal of neuroscience.

[16] Hynynen K, Colucci V, Chung A, Jolesz F (1996). Noninvasive arterial occlusion using MRI-guided focused ultrasound. Ultrasound in medicine & biology.

[17] Raymond SB, Skoch J, Hynynen K, Bacskai BJ (2007). Multiphoton imaging of ultrasound/Optison mediated cerebrovascular effects in vivo. Journal of cerebral blood flow and metabolism.

[18] Plamondon H, Khan S (2005). Characterization of anxiety and habituation profile following global ischemia in rats. Physiology & behavior.

[19] Milot M, Plamondon H (2008). Ischemia-induced hyperactivity: effects of dim versus bright illumination on open-field exploration and habituation following global ischemia in rats. Behavioural brain research.

[20] Morris RG, Garrud P, Rawlins JN, O'Keefe J (1982). Place navigation impaired in rats with hippocampal lesions. Nature.

[21] Zhang WN, Pothuizen HH, Feldon J, Rawlins JN (2004). Dissociation of function within the hippocampus: effects of dorsal, ventral and complete excitotoxic hippocampal lesions on spatial navigation. Neuroscience.

[22] Blanco JE, Anderson KD, Steward O (2007). Recovery of forepaw gripping ability and reorganization of cortical motor control following cervical spinal cord injuries in mice. Experimental neurology.

[23] Yang FY, Lu WW, Lin WT, Chang CW, Huang SL (2015). Enhancement of Neurotrophic Factors in Astrocyte for Neuroprotective Effects in Brain Disorders Using Low-intensity Pulsed Ultrasound Stimulation. Brain stimulation.

[24] Lin WT, Chen RC, Lu WW, Liu SH, Yang FY (2015). Protective effects of low-intensity pulsed ultrasound on aluminum-induced cerebral damage in Alzheimer's disease rat model. Scientific reports.

[25] Su WS, Tsai ML, Huang SL, Liu SH, Yang FY (2015). Controllable permeability of blood-brain barrier and reduced brain injury through low-intensity pulsed ultrasound stimulation. Oncotarget.

[26] Yang FY, Chang WY, Chen JC, Lee LC, Hung YS (2014). Quantitative assessment of cerebral glucose metabolic rates after blood-brain barrier disruption induced by focused ultrasound using FDG-MicroPET. Neuroimage.

[27] Yang FY, Lin YS, Kang KH, Chao TK (2011). Reversible blood-brain barrier disruption by repeated transcranial focused ultrasound allows enhanced extravasation. Journal of controlled release.

[28] Suarez M, Molina S, Rivarola MA, Perassi NI (2002). Effects of maternal deprivation on adrenal and behavioural responses in rats with anterodorsal thalami nuclei lesions. Life sciences.

[29] Fromm L, Heath DL, Vink R, Nimmo AJ (2004). Magnesium attenuates post-traumatic depression/anxiety following diffuse traumatic brain injury in rats. Journal of the American College of Nutrition.

[30] Pedraza C, Garcia FB, Navarro JF (2009). Neurotoxic effects induced by gammahydroxybutyric acid (GHB) in male rats. The international journal of neuropsychopharmacology / official scientific journal of the Collegium Internationale Neuropsychopharmacologicum.

[31] Suresh R, Ramesh Rao T, Davis EM, Ovchinnikov N, Mc Rae A (2008). Effect of diagnostic ultrasound during the fetal period on learning and memory in mice. Annals of anatomy.

[32] Han H, Ma Y, Eun JS, Li R, Hong JT, Lee MK, Oh KW (2009). Anxiolytic-like effects of sanjoinine A isolated from Zizyphi Spinosi Semen: possible involvement of GABAergic transmission. Pharmacology, biochemistry, and behavior.

